# Wurtzite AlGaAs Nanowires

**DOI:** 10.1038/s41598-020-57563-0

**Published:** 2020-01-20

**Authors:** L. Leandro, R. Reznik, J. D. Clement, J. Repän, M. Reynolds, E. V. Ubyivovk, I. V. Shtrom, G. Cirlin, N. Akopian

**Affiliations:** 1 DTU Department of Photonics Engineering, Technical University of Denmark, 2800 Kgs. Lyngby, Denmark; 2 ITMO University, Kronverkskiy pr. 49, 197101 St. Petersburg, Russia; 3 St. Petersburg State University, St. Petersburg, Russia; 4 St.Petersburg Academic University, RAS, St. Petersburg, 194021 Russia; 5 St. Petersburg Electrotechnical University “LETI”, Prof. Popova 5, St. Petersburg, 197376 Russia

**Keywords:** Materials science, Nanoscience and technology, Optics and photonics

## Abstract

Semiconducting nanowires, unlike bulk, can be grown in both wurtzite and zincblende crystal phases. This unique feature allows for growth and investigation of technologically important and previously unexplored materials, such as wurtzite *AlGaAs*. Here we grow a series of wurtzite *AlGaAs* nanowires with *Al* content varying from 0.1 to 0.6, on silicon substrates and through a comparative structural and optical analysis we experimentally derive, for the first time, the formula for the bandgap of wurtzite *AlGaAs*. Moreover, bright emission and short lifetime of our nanowires suggest that wurtzite *AlGaAs* is a direct bandgap material.

## Introduction

Polytypism^1^ is an exceptional property of nanowires and a new degree of freedom which enables the engineering of the electronic structure without change of material. For example, today’s atomically-precise control over the crystal-phase switching in nanowires^2,3^ allows to grow strain-free polytypic formations along the growth axis^4,5^, even small enough to form quantum dots^6,7^. The wurtzite phase is not observable at ambient conditions in bulk of any A^III^B^V^ materials except for nitrides, while it can be obtained in nanowires. For this property and its technological implications, a great deal of attention has been drawn, in recent years, to nanowires system from scientific community^8–10^. However, for designing of novel structures and devices, knowledge of bandgaps and band alignments of the different crystal phases of new materials is crucial.

In particular, *Al*_*X*_*Ga*_*1-X*_*As* nanowires provide a promising platform for fabrication of advanced devices. For example, adding the *Al* component to the widely studied *GaAs*^11,12^ allows to tune the emission in a wide range of wavelengths while, *AlGaAs*, having higher energy than *GaAs*, allows the combination of these two materials to fabricate strain-free quantum devices^13^.

However, the knowledge about wurtzite *AlGaAs* is limited in the literature^14–17^, and is mainly grown as a shell around wurtzite *GaAs* core^15,16^. Importantly, the bandgap of wurtzite *AlGaAs* was neither predicted theoretically nor measured experimentally.

In this work, we grow wurtzite *AlGaAs* nanowires, in a wide range of *Al* content *x*, and we present a comparative optical and structural study, empirically revealing the trend for the bandgap of wurtzite *Al*_*X*_*Ga*_*1-X*_*As*. We grow our samples by *Au*-catalyzed vapor-liquid-solid technique in a molecular beam epitaxy (MBE) reactor (see methods section for details) obtaining high crystalline quality structures with any chosen *Al* content.

## Results and Discussion

**Figure 1:**
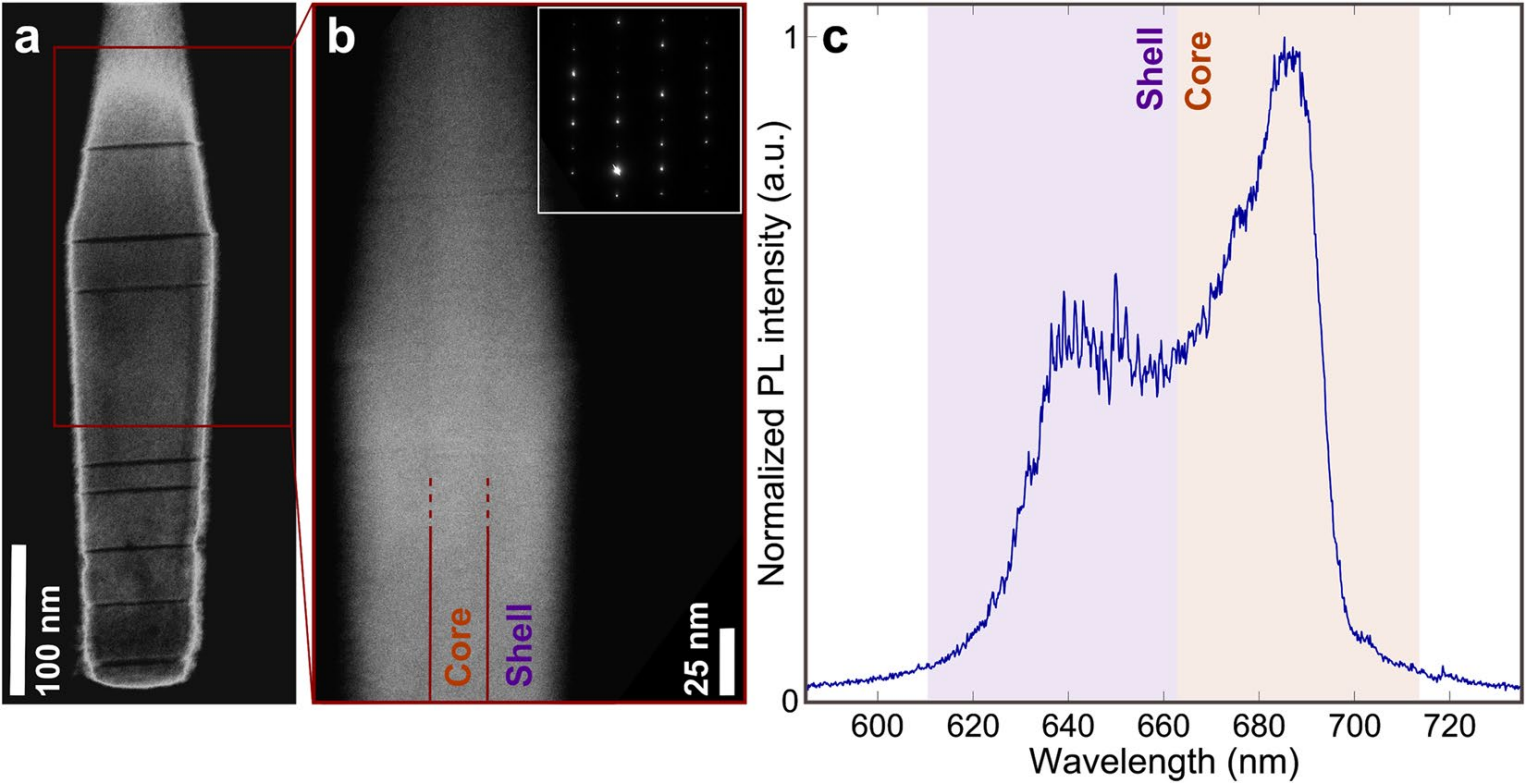
Structural analysis and photoluminescence of *AlGaAs* core-shell nanowires. (**a**) Transmission electron microscopy image of an *Al*_0.6_*Ga*_0.4_As nanowire showing only a few short zincblende insertions in otherwise pure wurtzite crystal structure. (**b**) Higher resolution measurement of the same nanowire in (**a**), with different microscopy parameters to enhance the visibility of the core-shell structure. Inset: diffraction pattern of the nanowire clearly showing pure wurtzite phase. (**c**) Scanning micro-photoluminescence measurement, showing the integrated emission of a large number of nanowires at 2 K. The spectrum shows two peaks that we attribute to shell and core emission.

In Fig. 1a,b, we show the transmission electron microscopy (TEM) images of a typical *Al*_0.6_*Ga*_0.4_*As* nanowire. In Fig. 1a, only a few short zincblende insertions, spontaneously formed during the nanowire growth, are visible as darker lines, consistent with our previous observation of a typical density of ~10–30 insertions/µm^13^. The zoom-in in Fig. 1b is optimized, during the TEM measurement, to resolve the inner part of the nanowire, highlighting the core-shell structure. The thick shell surrounding the core is masking the difference in the *Al* content between the core and the shell (Fig. 1a,b), resulting in a similar core-shell brightness in the images. Furthermore, the shell, protects and passivate^18^ the core surface. Given a certain *Al/Ga* ratio in the reactor, the nanowires grow with such core-shell formation spontaneously, where the core has lower *Al* content than the shell, and thus is expected to have lower bandgap. The origin of such growth has been studied in our previous work^17^, where the core-shell composition has been measured, via energy dispersive X-ray spectroscopy (EDX), to be always lower than nominal. The core grows with the diameter equal to the size of the catalyst droplet initially deposited on the substrate, which is kept constant during growth. Furthermore, TEM diffraction pattern analysis shows the nanowires to be always mostly pure wurtzite structure. The core-shell structure results in a double-peak emission, shown in the photoluminescence (PL) measurement in Fig. 1c. Here, the sample is excited above-bandgap and is scanned while continuously collecting micro-PL signal for 30 s, thus integrating the emission of large number of nanowires, on the order of thousands. This results in a measurement similar to macro-PL, referred here as scanning-PL (sPL). Because of the measured difference in *Al* content in the core and the shell, we attribute the short-wavelength and long-wavelength branches of the emission to shell and core, respectively. Further details on TEM, EDX and sPL can be found in our previous works^13,17^.

**Figure 2:**
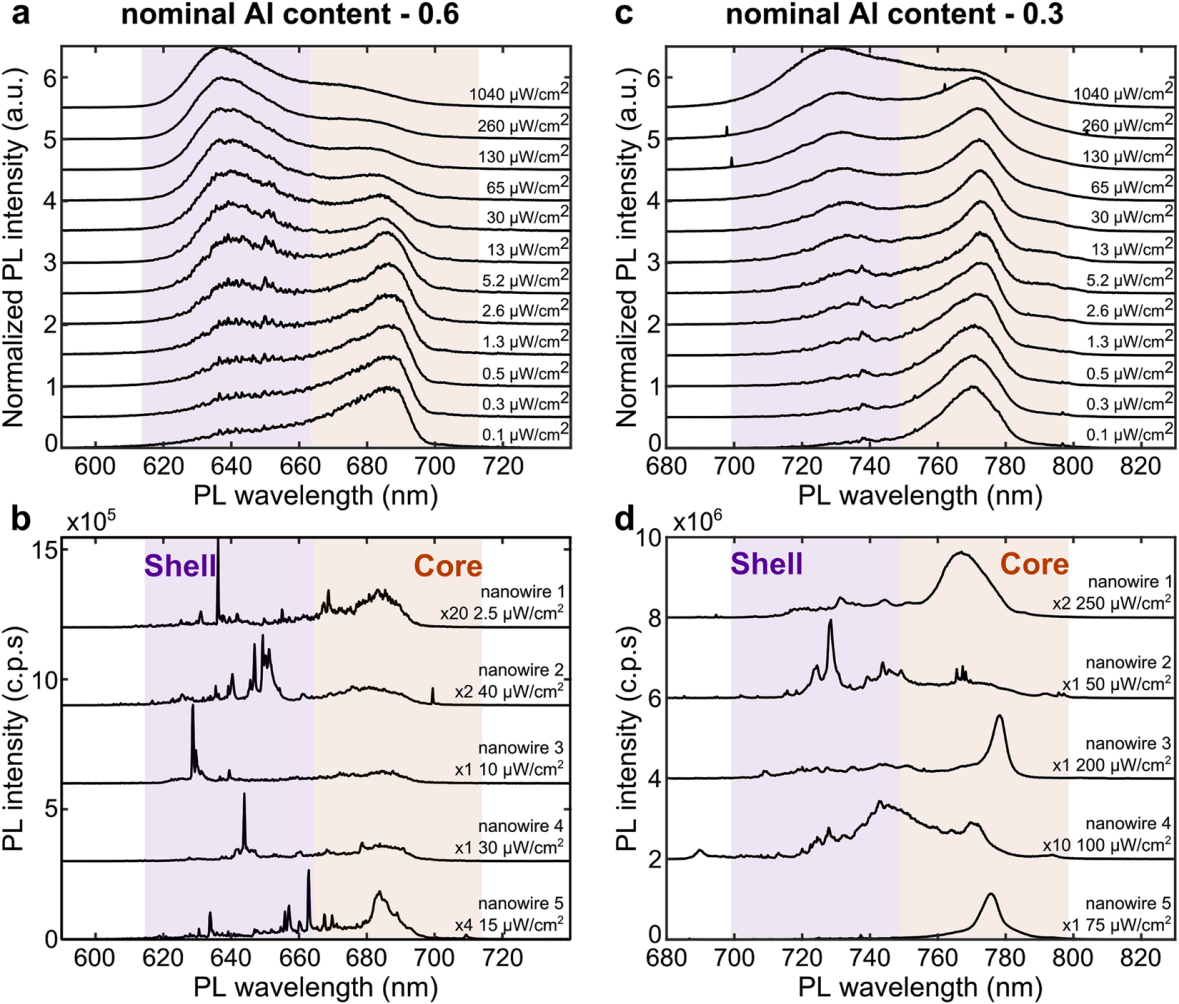
Excitation power dependence of nanowire emission and photoluminescence of single nanowires. (**a**) Scanning micro-photoluminescence showing the integrated emission of a large number of *AlGaAs* nanowires for different excitation powers, each normalized to the respective maximum. Nominal *Al* content is 0.6. (**b**) Micro-photoluminescence of 5 single nanowires on the same sample of (**a**), showing that the emission of an individual nanowire is generally composed of multiple sharp lines that we attribute to crystal-phase structures. (**c**,**d**) Same as (**a**,**b**) for nominal *Al* content of 0.3. All spectra are measured at 2 K and vertically shifted for clarity.

In Fig. 2a,c, we show the sPL measured at different excitation powers for samples with *Al* content of 0.6 and 0.3, respectively. The long-wavelength peak, attributed to the core, is dominant at low excitation power. With increasing excitation power, the shell states start to populate and emit, resulting in the emergence of a short-wavelength peak that eventually dominantes. We attribute this behavior to state-filling effects in the thin core (~20 nm) at high excitation powers. The excitation power dependent PL measurements (see Fig. S1 in the Supplementary Information) show this dynamics and also high brightness of our nanowires. We note that the data in Fig. 2a,c are normalized to their relative maxima and none of the peaks saturate. In Fig. 2b,d, we show the micro-PL for five different individual nanowires on each of the samples. This shows that the integrated emission measured with the sPL at low excitation powers is composed of a diverse and rich ensemble of individual nanowire peaks. These distributed peaks are attributed to crystal-phase structures in the nanowires with type-II band alignment^6,19^. These formations confine the charges and provide a radiative path for recombination. At the same time, they are consistently observed to be only a few atomic-layers thick, and thus are expected to have transition energies very close to the bandgap of *AlGaAs*. We note that previous works^20^ have highlighted the possibility of having random aggregates of *Al*-poor regions at the corners of hexagonal *GaAs/AlGaAs* structures, emitting light with similar spectral features to what observed in Fig. 2b,d. Our cross-sectional TEM measurements (not shown) do not reveal any trace of such compositional fluctuations and thus we attribute the emission to crystal phase structures, which are indeed observed in our TEM data.

**Figure 3:**
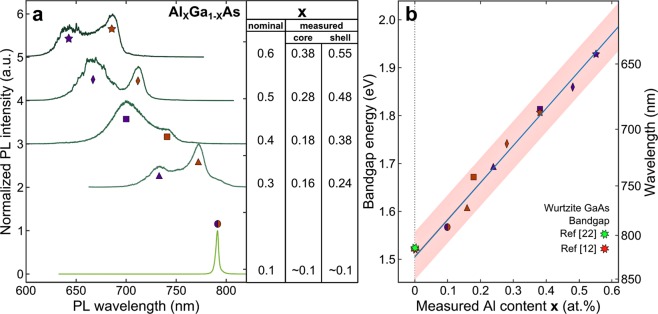
Experimental determination of wurtzite *Al*_*X*_*Ga*_*1-X*_*As* bandgap. (**a**) Integrated photoluminescence of a large number of *Al*_*X*_*Ga*_*1-X*_*As* nanowires for the nominal *Al* content *x* = 0.1, 0.3, 0.4, 0.5, 0.6. For each nominal content, the actual *Al* content in core and shell has been measured using transmission electron microscopy^21^. All spectra are vertically shifted for clarity, and normalized to their respective maxima. (**b**) Peak emission energy plotted as a function of the measured *Al* content for core and shell. Measurements on nominal *Al* content of 0.1, 0.3, 0.4, 0.5 and 0.6, correspond to circles, triangles, squares, diamonds and stars, respectively, while orange and purple correspond to core and shell emission, respectively. The data are fitted with a linear model which results in the blue line (coefficient of determination R^2^ = 0.964). The measured values of wurtzite *GaAs* bandgap reported in literature are added to the graph for comparison, and are indicated by a red^12^ and a green^22^ stars. The light-red shadow identifies the estimated highest uncertainty of ~0.1 in *Al* content due to statistical fluctuations of the growth parameters, that results in variation of the peak emission energy from nanowire to nanowire.

By measuring the nanowire ensemble emission (such as shown in Fig. 2a,c) for samples with different *Al* contents, we obtain the dependence of the nanowire emission energy on the actual *Al* content and thus experimentally determine the bandgap of wurtzite *Al*_*X*_*Ga*_*1-X*_*As* for 0.1 < *x* < 0.6. In Fig. 3a we show the integrated emission of a large number of nanowires, for different *Al* contents. All the spectra show two peaks, due to core and shell emission, except for the sample with nominal *Al* 0.1. In this case the composition of both the core and the shell is very close to the nominal. This results in a non-distinguishable core in TEM observations and in only one peak in the emission spectrum. We note that at low excitation power, crystal-phase structures can prevent the appearance of band-to-band transitions in the emission spectra, as photo-generated carriers fall into the lower energy states in the crystal-phase dots. However, we consistently observe that crystal phase insertions are very small in size — a few monolayers only (see Fig. S2 in the Supplementary Information). Thus the energy levels in such structures are very close to the band-to-band transition energies of the host material. Macro-PL, on the other hand, showing the average distribution of all the emission lines, is clearly grouped in two peaks — core and shell. We thus have used macro-PL (or sPL) data for the derivation of *AlGaAs* bandgap.

The position of the maxima in the spectra in Fig. 3a is evaluated in respect to the actual content of *Al* measured via EDX^17,21^, and is shown in Fig. 3b, revealing the linear trend. In this case, the shell and core emission peak energies are plotted against different values of *x*. The values of measured *Al* composition and corresponding energy of emission are summarized in Table 1. The agreement of the two peak emission with a linear trend confirms our hypothesis for their origin from shell and core. A linear regression provides the empirical formula for the bandgap of wurtzite *Al*_*X*_*Ga*_*1-X*_*As* to be:

$${{\rm{E}}}_{{\rm{g}}}=1.506\,{\rm{eV}}+{\rm{x}}\ast 0.777\,{\rm{eV}},\,{\rm{where}}\,0 < {\rm{x}} < 0.6$$

with a remarkable coefficient of determination of the fit, R^2^ = 0.964. This empirical trend provides, by extrapolation down to *x* = 0, a value for the bandgap of pure wurtzite *GaAs* of 1.51 eV, in good accordance with the experimental values found in the literature: 1.52 eV at 7 K^22^, and 1.517 eV at 10 K^12^. We also note no evident change of the slope, known for zincblende *AlGaAs* at *Al* content of ~0.4 due to the bandgap transition from direct to indirect^23^. The lack of such transition for the wurtzite phase suggests to extend the range of emission wavelength that can be reached with *AlGaAs*. Furthermore, we note that even if the experimental data follows a linear trend for 0.1 < x < 0.6, the bandgap is not necessarily linear. Thus linear extrapolations of our formula above *x* = 0.6 might not lead to correct predictions. Comparison with current predictions^24^ and experiments^25^ indeed suggests the non-linearity of the bandgap. In Fig. 3b, we have also included an estimated uncertainty of 0.1 in *Al* content by means of a red shade. This uncertainty is the estimated maximum uncertainty in Al content due to statistical fluctuations of the growth parameters, resulting in small variations of *Al* content from nanowire to nanowire, as we observe in our TEM measurements.

**Table 1:** Aluminum content measured in the nanowires and resulting band-gap.

Measured *Al* content ***x*** (at.%)	0.1	0.16	0.18	0.24	0.28	0.38	0.38	0.48	0.55
Bandgap **E**_**g**_ (eV)	1.568	1.606	1.671	1.692	1.742	1.808	1.813	1.859	1.929

**Figure 4:**
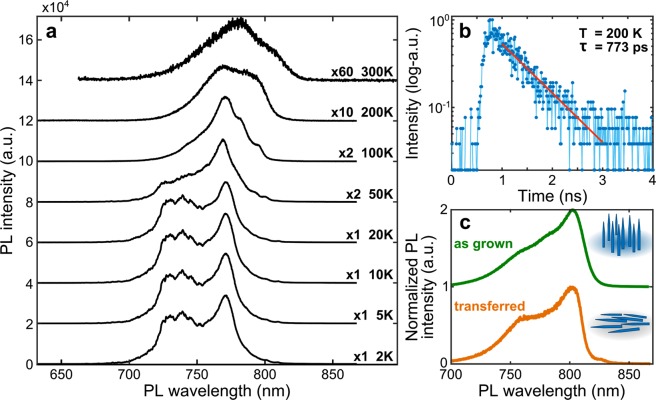
Photoluminescence temperature dependence, lifetime and photoluminescence of transferred nanowires. (**a**) Macro-photoluminescence of *Al*_*0*.*3*_*Ga*_*0*.*7*_*As* nanowires at different temperatures under the same excitation conditions. All spectra are vertically shifted and multiplied by an arbitrary factor for clarity. (**b**) Lifetime measurement at 200 K of a narrow spectral region around 770 nm, showing fast decay. The experimental data points (blue circles) are fitted with a mono-exponential decay (red line), revealing a decay constant τ = 773 ± 17 ps. (**c**) Room temperature normalized macro-PL spectrum of as-grown *Al*_0.3_*Ga*_0.7_*As* nanowires compared to nanowires transferred to a silicon-dioxide substrate. The similar spectra show that the measured emission comes from the nanowires and not from any layers residual from growth. The spectra are vertically shifted for clarity.

In Fig. 4a we show the temperature dependence of the photoluminescence of a *Al*_*0*.*3*_*Ga*_*0*.*7*_*As* nanowire ensemble. Our nanowires show pronounced emission at room temperature, even though the intensity drops in the range 200–300 K. The wavelength of emission does not have a noticeable change in the temperature range from 2 K to about 100 K, and undergoes a red-shift at higher temperatures. This behavior is expected from the Varshni empirical equation for the temperature dependence of semiconductor bandgaps^26^ and is similar, for instance, to the wurtzite *GaAs*^22^. The measurements at low temperatures (2–50 K) show a notched spectrum, composed of several sharp features, that we attribute to bright emission from crystal-phase structures collected during the scan at these temperatures. These sharp features disappear at higher temperatures, leaving smooth spectra. We associate this to the escaping of charges from the trapping crystal-phase structures due to thermal energy, allowing for emission from the *AlGaAs* bandgap transition.

In Fig. 4b, we show the lifetime measurement for the emission peak at 770 nm at 200 K. The fit to a monoexponential decay reveals a fast decay with lifetime τ = 773 ± 17 ps, similar to the room temperature decay rate of *GaAs*^27^. These results further suggest that the emission at high temperature originates from the direct bulk-like emission of wurtzite *AlGaAs* and not from crystal-phase related emission, which is expected to have spatially indirect transitions with slower recombination times^6^. Although the actual lifetime in our measurement can be masked by non-radiative recombinations expected at this temperature, together with high brightness of the nanowires it suggests that wurtzite *AlGaAs* is a direct bandgap semiconductor for *Al* content less than 0.6.

Finally, in order to exclude a possible emission from the *AlGaAs* layer in between nanowires, we transfer the nanowires on a clean silicon-dioxide substrate. In Fig. 4c, we compare the emission spectra from an ensemble of as-grown and transferred nanowires. The clear similarity of the spectra leads to conclude that none of the emission studied in this work is due to the remnant *AlGaAs* layer.

In summary, we have experimentally derived a formula for the bandgap of wurtzite *AlGaAs* for aluminum contents up to 0.6. Our results show that the bandgap, within that range, follows a linear trend and agrees remarkably well with known experimental values for *Al* content of 0, i.e. wurtzite *GaAs*. We reported a nearly-pure crystalline structure, via TEM observations, and showed its wide tunability range of the emission wavelength via PL measurements. Finally, we have shown the spectra of the emitted light as a function of sample temperature, observing the expected trend for bandgap of bulk semiconductors. High brightness and short lifetime of our nanowires indicate that the bandgap of Wurtzite *AlGaAs* is direct for *Al* content less than 0.6, although an unambiguous proof is yet to be demonstrated.

## Methods

### Sample growth

Our nanowires are grown by *Au*-catalyzed vapor-liquid-solid (VLS) method in a molecular beam epitaxy reactor on a Si(111) substrate. First, an Au layer 0.1 nm thick is deposited at 550 °C. The gold forms droplets of ~20 nm in diameter which define the size of the core of the nanowires. *Al*, *Ga* and *As* molecular beams are applied simultaneously to grow *Al*_*X*_*Ga*_*1-X*_*As* nanowires at 510 °C. The ratio between the *Al* and *Ga* defines the nominal *Al* content x. MBE growth of *Au*-catalyzed *AlGaAs* nanowires leads to spontaneous formation of an unintentional core-shell structure. The shell forms via vapor-solid (VS) process, whereas the core via VLS mechanism, typically with *Al* content larger than in the core^17^.

### Photoluminescence setup

The optical measurements have been performed in a closed-cycle He cryostat. A continuous wave laser at 532 nm was used for above bandgap excitation for all photoluminescence measurements, while a 400 nm pulsed laser (temporal pulse full width half maximum ~90 ps) was used for lifetime measurements. Scanning-PL measurements have been performed in a micro-PL configuration, scanning in a straight line for 30 s on the sample kept at 2 K (except for temperature dependent PL measurements). The spectrum is measured using a low-noise charge-coupled device connected to a 75 cm long spectrometer. The same setup, has been used for micro-PL measurements of individual nanowires. Macro-PL measurements have been done using a similar setup, with a lens with larger focal length, without any scanning. The lifetime is measured with a fast single-photon detector (~50 ps jitter) combined with time-tagging electronics (~100 ps jitter).

## Supplementary information

Supplementary Information.

## Data Availability

The data supporting the findings of this study are available from the corresponding author on reasonable request.
