# Supplementary material for: Wurtzite AlGaAs Nanowires
Source: Sci Rep. 2020 Jan 20;10:735. doi: 10.1038/s41598-020-57563-0 (PMC6971003; doi:10.1038/s41598-020-57563-0)
Supplement: Supplementary file 1 — Supplementary Information. [file 41598_2020_57563_MOESM1_ESM.pdf]

Supplementary information for
Wurtzite AlGaAs Nanowires

L. Leandro[1], R. Reznik[2], J.D. Clement[1], J. Repän[1], M. Reynolds[1], E.V. Ubyivovk[3], I. V. Shtrom[3,4], G. Cirlin[2,4,5] and N. Akopian[1]*

[1]DTU Department of Photonics Engineering, Technical University of Denmark, 2800 Kgs. Lyngby, Denmark
[2]ITMO University, Kronverkskiy pr. 49, 197101 St. Petersburg, Russia
[3]St. Petersburg State University, St. Petersburg, Russia
[4]St.Petersburg Academic University, RAS, St. Petersburg 194021, Russia
[5]St.Petersburg Electrotechnical University “LETI”, Prof. Popova 5, St. Petersburg 197376, Russia
*e-mail: nikaak@fotonik.dtu.dk

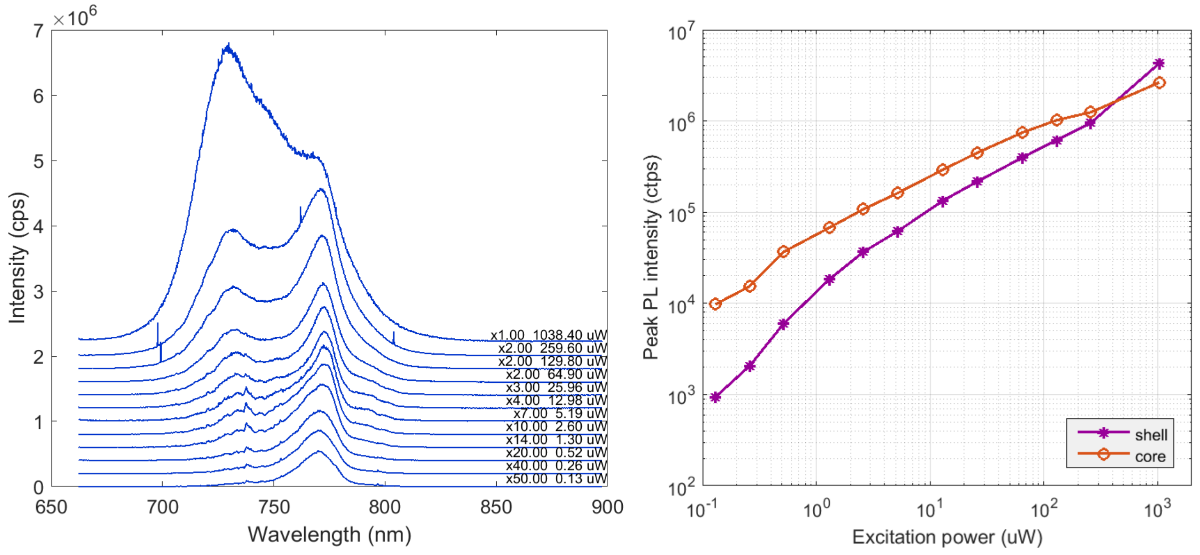

(a) **(b)**

Figure S1 | sPL of a typical *$Al_{0.3}Ga_{0.7}As$* nanowire under different excitation intensities. a) Spectra measured at different excitation laser intensities and **b)** peak power of the peaks attributed to shell (short wavelenght peak) and core (long wavelength peak), respectively.

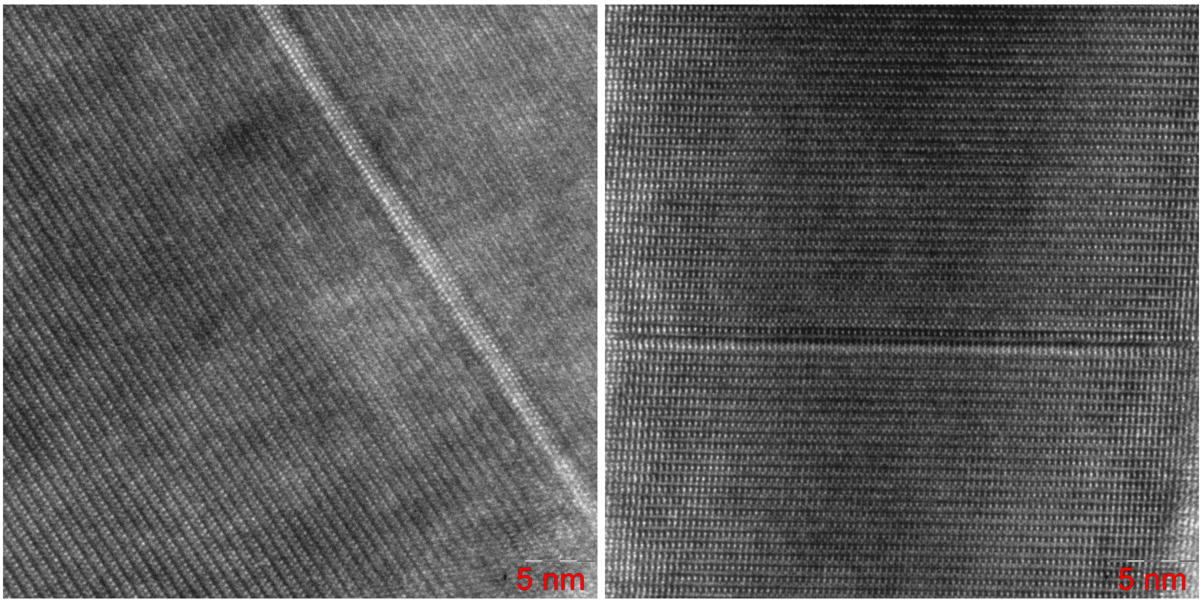

Figure S2 | TEM measurements of typical Zincblende insertions or stacking faults in two different Wurtzite *AlGaAs* nanowires, showing thicknesses of 2-3 monolayers (i.e. 4-6 atomic layers).
